# Supplementary material for: Facile Generation of Potent Bispecific Fab via Sortase A and Click Chemistry for Cancer Immunotherapy
Source: Cancers (Basel). 2021 Sep 10;13(18):4540. doi: 10.3390/cancers13184540 (PMC8467688; doi:10.3390/cancers13184540)
Supplement: Supplementary file 1 [file cancers-13-04540-s001.zip › cancers-1361297-supplementary.pdf]

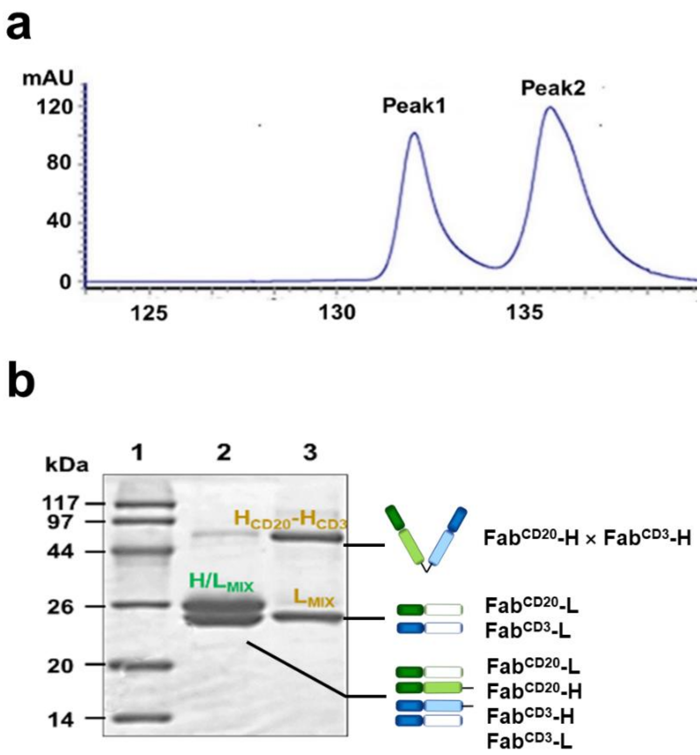

Figure S1. (a) Size exclusion chromatography (SEC) purification of BiFab$^{CD20/CD3}$. (b) SDS-PAGE analysis of peaks from (a); Line 1, high molecular weight protein marker; Line 2, reduced protein product from peak 1; Line 3, reduced protein product from peak 2.

a

Cell line	MFI		Corrected MFI
	+	−	
Ramos	114	5.8	19.6
Raji	48.6	0.6	81
Daudi	43.4	0.7	62
K562	0.7	0.7	-

b

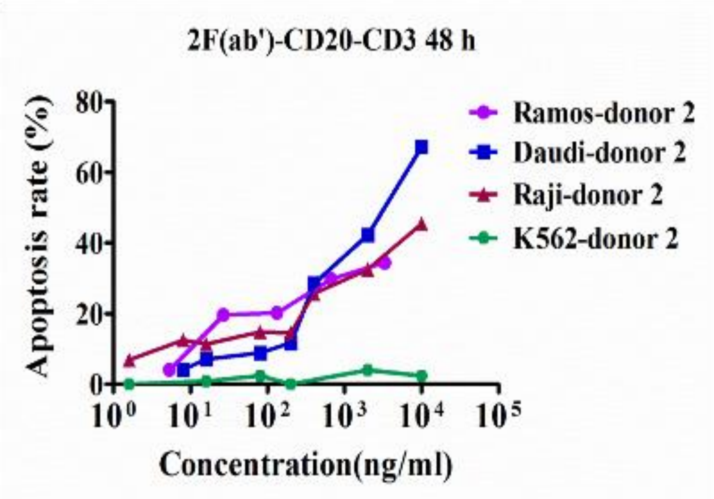

Figure S2. BiFab$^{CD20/CD3}$ activated T cells in the presence of target cell lines with different antigen expression level and mediated target cells killing in a T cell-dependent manner. (a) Target cell lines with different antigen expression level are measured by flow cytometry; (b) Target cell lines of different CD20 expression level and PBMC isolated from a healthy donor (1: 5 cell ratio) were incubated with serial concentrations of BiFab$^{CD20/CD3}$ for 48 h.
